# EI24 binds to IGF1R, enhancing glucose homeostasis and fostering healthy aging in male mice

**DOI:** 10.3389/fragi.2025.1564730

**Published:** 2025-06-10

**Authors:** You-Min Kim, Seung Eon Lee, Yaechan Song, Tae Wook Nam, Jaehoon Lee, Je Kyung Seong, Wan Namkung, Han-Woong Lee

**Affiliations:** ^1^ Department of Biochemistry, College of Life Science and Biotechnology, Yonsei University, Seoul, Republic of Korea; ^2^ Gemcro, Inc., Seoul, Republic of Korea; ^3^ Korea Model animal Priority Center (KMPC), Seoul National University, Seoul, Republic of Korea; ^4^ Laboratory of Developmental Biology and Genomics, Research Institute for Veterinary Science, and BK21 PLUS Program for Creative Veterinary Science Research, College of Veterinary Medicine, Seoul National University, Seoul, Republic of Korea; ^5^ College of Pharmacy, Yonsei Institute of Pharmaceutical Sciences, Yonsei University, Incheon, Republic of Korea

**Keywords:** EI24, aging, GLUT4, IGF1R, diabetes mellitus

## Abstract

**Introduction:**

The etoposide-induced 2.4 kb transcript (EI24) plays a crucial role in autophagy, facilitating the clearance of damaged proteins and organelles to maintain cellular homeostasis. While autophagy is widely recognized for its beneficial effects on healthy aging, the effects of EI24 overexpression remain unclear.

**Methods:**

We analyzed the interaction of EI24 with the insulin-like growth factor 1 receptor (IGF1R), a key molecule associated with aging. *Ei24* transgenic (TG) mice were generated to assess the effects of Ei24 overexpression on aging, glucose homeostasis, and resistance to streptozotocin (STZ)-induced diabetes.

**Results:**

EI24 was found to bind to IGF1R, specifically engaging with its transmembrane (TM) domain near the cytoplasmic membrane, and suppress its phosphorylation. Male *Ei24* TG mice exhibited signs of healthier aging, with reduced aging markers in the kidney, liver, and pancreas. Moreover, Ei24 overexpression enhanced glucose uptake, likely due to increased Glut4 expression in muscle tissue. *Ei24* TG mice also demonstrated resistance to high-dose STZ-induced diabetes.

**Conclusion:**

These findings suggest that Ei24 overexpression contributes to improved glucose regulation and healthier aging across multiple organs. By interacting with IGF1R, EI24 may provide a novel mechanism for promoting metabolic and age-related health.

## 1 Introduction

The insulin-like growth factor 1 receptor (IGF1R) serves as a key regulator of growth, development, and the multifaceted processes of aging (Park et al., 2022). Although reduced IGF1R signaling is often associated with beneficial effects on longevity, its activation remains critical in specific organs, demonstrating its context-dependent and tissue-specific roles (Gubbi et al., 2018). Notably, several long-lived mouse models—including Ames dwarf, Snell dwarf, and Ghr knockout mice—exhibit markedly reduced circulating IGF1 levels, highlighting the inverse association between IGF1 signaling and lifespan (Hager et al., 2024; Brown-Borg and Bartke, 2012). In addition, mice heterozygous for IGF1R deletion show extended lifespan, particularly in females, further supporting the role of IGF1R inhibition in longevity promotion (Holzenberger et al., 2003). On the other hand, IGF1R is also crucial for tissue integrity; for instance, global IGF1R knockout mice display impaired skeletal muscle development, characterized by reduced myofiber number and size (Mavalli et al., 2010). Muscle-specific Igf1r depletion enhances glucose transporter type 4 (Glut4) transcription, promoting glucose uptake and metabolic regulation in the muscle (O'Neill et al., 2015), highlighting the role of IGF1R in maintaining metabolic homeostasis and energy balance within the muscle tissue.

Aging, a universal phenomenon in all living organisms, has long captivated scientific inquiry, fueling efforts to extend human longevity and promote a healthier lifespan. This inevitable process disrupts tissue homeostasis and leads to physical and cognitive decline, with cellular deterioration tied to chronic disorders such as diabetes mellitus (DM) (DiLoreto and Murphy, 2015; Melzer et al., 2020). By preserving cellular equilibrium, autophagy functions as a critical mechanism that degrades and recycles impaired proteins and organelles, thereby supporting healthy aging (Aman et al., 2021). The proteins involved in autophagy are pivotal in the aging process and in the development of age-related diseases (Aman et al., 2021). It is therefore important to understand their functions and explore their therapeutic potential to delay age-related conditions.

The etoposide-induced 2.4 kb transcript (EI24), a downstream target of p53, is upregulated in response to the DNA damage induced by the chemotherapeutic agent etoposide, where it plays a crucial role in facilitating apoptosis (Gu et al., 2000). EI24 has also been implicated in the regulation of autophagy through its interaction with E3 ligases containing the RING domain (Devkota et al., 2016). EI24’s functions in cancer biology are highly context-dependent. For example, it acts as a tumor suppressor in pancreatic ductal adenocarcinoma and triple-negative breast cancer by inhibiting cell proliferation but promotes tumor growth in skin cancer (Zang et al., 2018; Li et al., 2017; Devkota et al., 2012). Depletion of Ei24 in pancreatic β-cells triggers apoptosis through ATPase sarcoplasmic/ER Ca^2+^-transporting 2 (Atp2a2)-mediated calcium dysregulations, which is driven by the interaction between Ei24 and Atp2a2 (Yuan et al., 2018). These findings suggest that the overexpression of EI24 may provide protection against the apoptosis triggered by such pathways. Indeed, the overexpression of Ei24 in transgenic (TG) mice has been demonstrated to confer limited protection against colorectal cancer growth (Nam et al., 2019). These findings emphasize the need for further investigation into EI24’s broader protective roles, especially in aging.

In this study, by examining the interaction between EI24 and IGF1R, we aimed to understand its effects on muscle Glut4 expression and glucose regulation. Given the crucial roles of both EI24 and IGF1R in maintaining tissue homeostasis, we also investigated the aging processes and glucose tolerance in Ei24 TG mice to explore these potential links.

## 2 Materials and methods

### 2.1 Cell culture and transfection

293T and C2C12 cells were purchased from the American Type Culture Collection. All cells were cultured and maintained in Dulbecco’s modified Eagle’s medium (SH30243.01; HyClone Laboratories, Inc.) supplemented with 10% fetal bovine serum (SH30919.03; HyClone Laboratories, Inc.) and 1% penicillin/streptomycin as described previously (Devkota et al., 2016). The 293T cells were transfected at 60%–70% confluency with 1 µg of EI24-HA-tagged DNA or 3 µg of IGF1R-flag-tagged DNA. OmicsFect™ *in vitro* transfection reagent (Omics Bio, CP2101) was added 24 h after seeding. The medium was changed at 1 h post-transfection.

### 2.2 Preparation of MEF cell lines

Embryos from Ei24 transgenic mice and wild-type (WT) mice were isolated between E13.5 and E14.5. The embryos were minced and typsinized for 20 min, and then seeded into T-75 cell culture dishes in 15 mL of Dulbecco’s modified Eagle’s medium (SH30243.01; HyClone Laboratories, Inc.). The cells were split at 1:2–1:3 ratios when freshly confluent, passaged two or three times to obtain a morphologically homogenous culture, and then frozen or expanded for further studies.

### 2.3 Protein extraction, immunoprecipitation, and Western blot analysis

Protein extraction, immunoprecipitation, and Western blotting were performed as previously described (Devkota et al., 2016; Nam et al., 2019), with minor modifications. Cells were lysed in ice-cold Triton X-100 lysis buffer, and tissues were homogenized in RIPA lysis buffer with a protease inhibitor mixture. The membranes were then probed with the primary antibodies (Supplementary Table S1).

### 2.4 Immunofluorescence (IF)

Immunofluorescence (IF) staining was performed as described previously (Devkota et al., 2016), with minor modifications. For frozen tissue sections, we used the same protocol as for cell IF staining, except for the blocking step, which utilized a different blocking buffer (5% goat serum in 0.5% triton X-100). Immunofluorescence staining was observed using a fluorescence microscope (Confocal Microscope LSM980, Carl Zeiss). The samples were then probed with the primary antibodies (Supplementary Table S1), and the nuclei were stained with DAPI (Abcam, Ab104139). The intensity of colocalization of EI24 and IGF1R signals was quantified using ImageJ software, with the intensity scale set at 5–50.

### 2.5 Animals and survival analysis

All animal care and experiments were conducted with the ethical approval of the Institutional Animal Care and Use Committee of Yonsei University (IACUC, 201711-656-02). The mice were housed in a specific pathogen-free facility at the Yonsei Laboratory Animal Research Center. *Ei24* TG mice were generated as described previously (Nam et al., 2019) and maintained on a C57BL/6J background. Survival analysis was performed using Kaplan–Meier curves to compare the experimental groups. Survival curves were plotted using GraphPad Prism version 5.0.2. The hazard ratio (HR) was calculated using the Cox proportional hazards model.

### 2.6 Senescence β-galactosidase staining (SA-β-gal)

Frozen tissue samples were stained with senescence β-galactosidase staining solution (Cell Signaling, #9860) as described previously (Cai et al., 2020). Following staining, tissues were washed with PBS and counterstained with eosin. The slides were examined under the ×20 objective of a Nikon Eclipse-80i microscope. The intensity of β-galactosidase staining was quantified using ImageJ software, with the intensity scale set at 0–135.

### 2.7 Glucose tolerance test (GTT) and insulin tolerance test (ITT)

Glucose tolerance tests (GTT) and insulin tolerance tests (ITT) were performed as described previously (Yuan et al., 2018). Mice were fasted overnight before undergoing GTT. Glucose (1.5 g/kg body weight) was administered intraperitoneally, and blood glucose levels in the tail vein blood were measured at 0, 30, 60, 90, 120, and 180 min using a glucometer (BAROZENII, GM01IAC). For the ITT, the mice were fasted for 6 h prior to the intraperitoneal injection of insulin (0.75 U/kg body weight). Their blood glucose levels were then measured postinjection at the timepoints listed above.

### 2.8 Reverse transcription-quantitative polymerase chain reaction (RT-qPCR)

RNA isolation and RT-qPCR were conducted as previously described (Nam et al., 2019) using TRIzol reagent (Invitrogen), a RevertAid First Strand cDNA Synthesis Kit (Thermo Fisher Scientific), and CFX (Bio-Rad) with primers (Supplementary Table S2).

### 2.9 Myoblast differentiation

To induce differentiation, C2C12 myoblasts were grown to confluence and then switched to a differentiation medium containing Dulbecco’s Modified Eagle’s Medium supplemented with 2% horse serum (Zhang et al., 2020). Cells were allowed to differentiate for up to 6 days, and the medium was changed every 48 h.

### 2.10 Diabetes induction and animal monitoring

Streptozotocin (STZ; Sigma) was dissolved in citrate buffer and then administered intraperitoneally at a dose of 200 mg/kg to 8-week-old male mice (Han and Liu, 2010). Mice with blood glucose levels of ≥300 mg/dL 2 days postinjection were considered to have STZ-induced diabetes. Blood glucose levels were monitored using a glucometer (GlucoDr. PLUS, AGM-3000).

### 2.11 Immunohistochemistry (IHC)

Pancreatic tissues were stained with insulin (Supplementary Table S1) using an IHC Application Solutions Kit (Cell Signaling, 13079) as described previously (Zang et al., 2018). Images were captured using the ×40 objective of a Nikon Eclipse-80i microscope. The intensity of insulin IHC staining was quantified using ImageJ software, with the intensity scale set at 0–170.

### 2.12 Statistical analysis

Data were analyzed using GraphPad Prism software. Survival data were analyzed using the log-rank (Mantel–Cox) test. The GTT and ITT results were presented as the mean ± SEM. Differences were assessed using two-way ANOVA and Bonferroni *post hoc* tests for multiple comparisons. A *p* value of <0.05 was considered statistically significant.

## 3 Results

### 3.1 EI24 directly interacts with IGF1R

**FIGURE 1:**
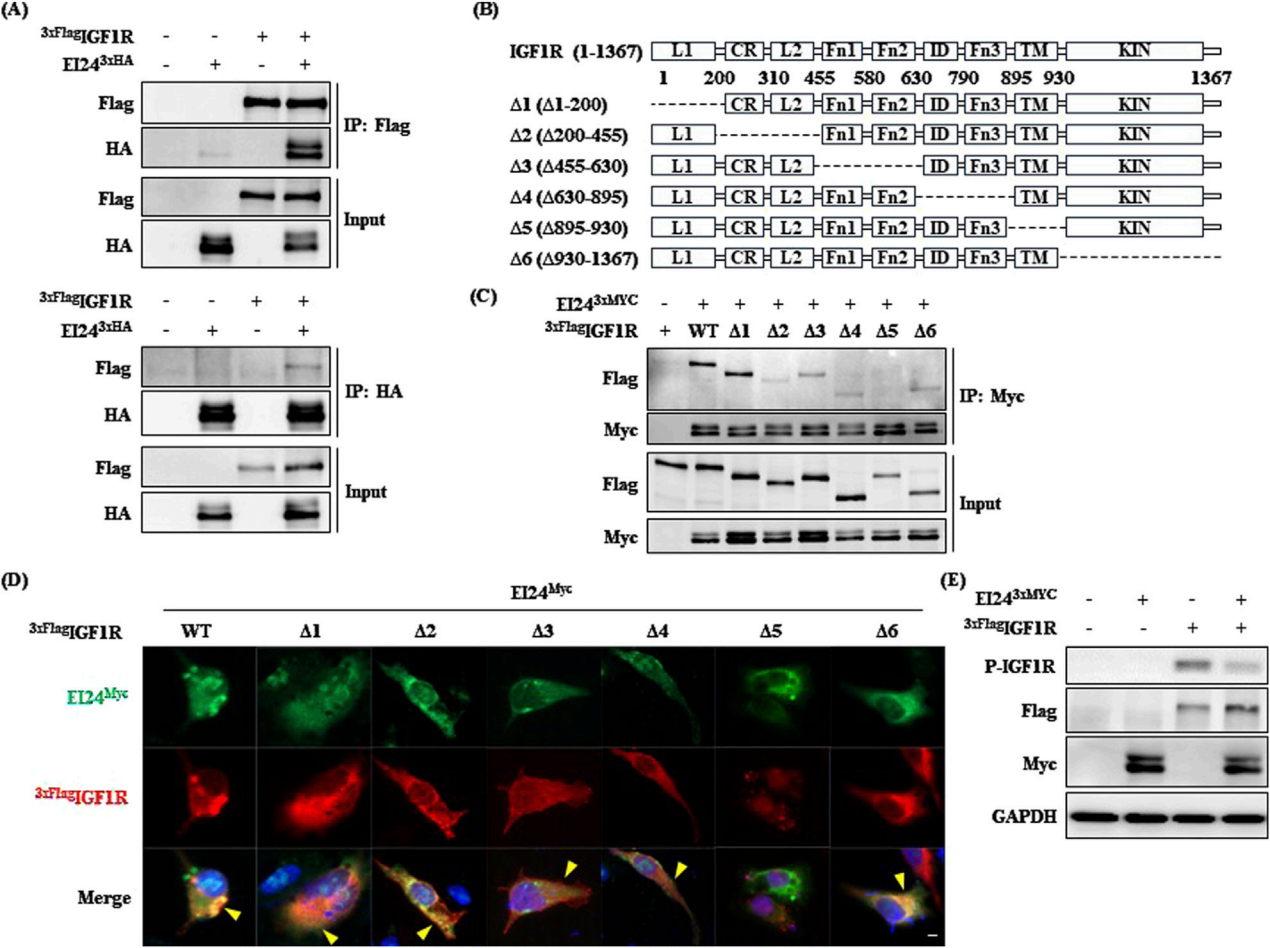
Etoposide-induced 2.4 kb transcript (EI24) binds to insulin-like growth factor 1 receptor (IGF1R). **(A)** Coimmunoprecipitation (Co-IP) and Western blotting analyses demonstrating the interaction between 3xFlag-tagged IGF1R and EI24-3xHA in transfected HEK 293T cells. Input refers to total cell lysates. **(B)** Schematic representation of IGF1R (1–1,367) and its deletion mutants (Δ1–Δ6) utilized in this study. The key domains of IGF1R, including L1, CR, L2, Fn1, Fn2, ID, Fn3, TM, and KIN, are indicated. **(C)** Co-IP analysis showing the interaction between EI24-3xMyc and 3xFlag-tagged IGF1R WT and deletion mutants. Lysates were immunoprecipitated with the Flag antibody, and Myc- and Flag-tagged proteins were detected by Western blotting. **(D)** Immunofluorescence illustrating the localization of EI24 (green, Myc-tagged) and IGF1R (red, Flag-tagged) in transfected cells. DAPI-stained nuclei (blue; scale bar = 10 µm). **(E)** Western blotting analysis validating the phosphorylation of IGF1R by EI24 in transfected HEK 293T cells.

In a previous mass spectrometry analysis, we identified an interaction between the insulin-like growth factor 2 receptor (IGF2R) and EI24 (Bahk et al., 2010). The BioGRID database also indicates a potential IGF1R–IGF2R interaction, adding complexity to IGF signaling (Han et al., 2017). IGF1R is a major factor in metabolic and aging-related pathways, significantly impacting energy metabolism and muscle development, while IGF2R primarily facilitates lysosomal enzyme trafficking without catalytic activity (Barclay et al., 2019; Vitale et al., 2019; LeRoith et al., 2021). Given the broad impact of IGF1R in aging processes, understanding how EI24 interacts with IGF1R is crucial to unraveling their combined role in the aging mechanism. Our coimmunoprecipitation (Co-IP) assays confirmed a direct interaction between EI24 and IGF1R (Figure 1A). Using the deletion constructs of IGF1R, we found that the transmembrane (TM) domain (Δ5) is essential for its interaction with EI24, as deletion of this domain significantly disrupted binding (Figures 1B,C). Immunofluorescence staining further supported these findings, revealing colocalization of EI24 and IGF1R predominantly in the cytoplasm and/or membrane periphery, except in cells expressing the Δ5 construct (Figure 1D; Supplementary Figure S1A). Moreover, coexpression of EI24 with IGF1R led to reduced phosphorylation of IGF1R compared with IGF1R expression alone (Figure 1E). Consistently, time-course analysis of IGF1-induced IGF1R phosphorylation in wild-type (WT) and Ei24 TG MEFs showed lower phosphorylation levels in TG MEFs, whereas AKT phosphorylation did not differ substantially between the groups (Supplementary Figure S1B, C). This finding suggests that EI24 binding may suppress IGF1R phosphorylation, thereby modulating its activity in processes such as aging and glucose metabolism.

### 3.2 Ei24 overexpression extends survival and reduces senescence in male mice

**FIGURE 2:**
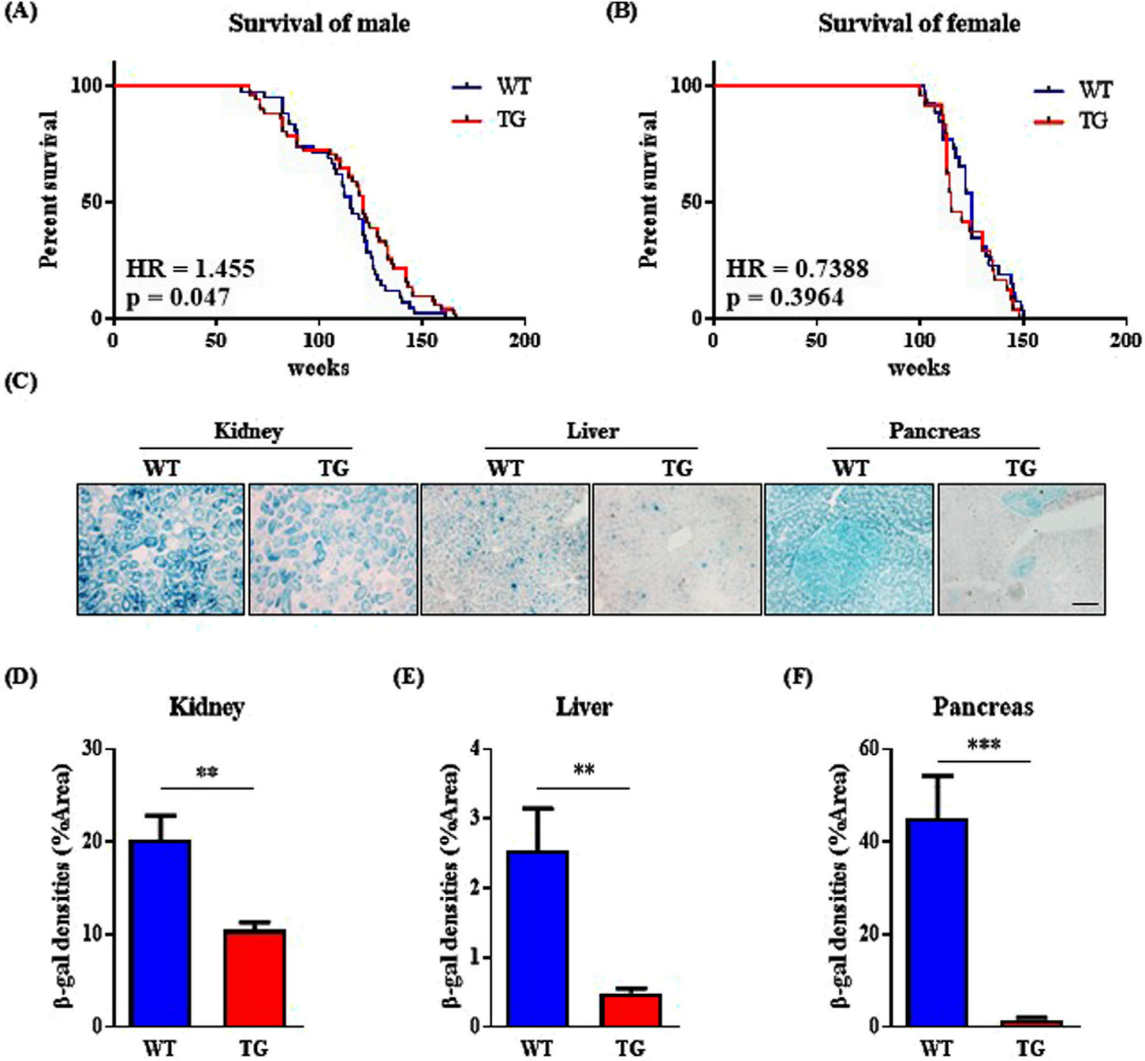
Elevated expression of *Ei24* prolongs lifespan and diminishes senescence in male mice. **(A)** Kaplan–Meier survival curves illustrating the percentage survival of WT and *Ei24* TG male mice (WT n = 30, TG n = 36). **(B)** Kaplan–Meier survival curves for WT and TG female mice (WT n = 26, TG n = 24). **(C)** Representative images of SA-β-gal staining (blue) in the kidney, liver, and pancreas of 75-week-old male WT and TG mice (scale bar = 100 µm). **(D–F)** Quantification of SA-β-gal positive cells as a percentage of the area in WT and TG kidneys, livers, and pancreas. (***p < 0.001, kidney: WT n = 6, TG n = 6; liver: WT n = 6, TG n = 6; pancreas: WT n = 6, TG n = 6).

We observed that EI24 overexpression inhibited IGF1R activation (Figure 1E; Supplementary Figures S1B, C), prompting us to question whether this suppression contributed to the lifespan extension reported in IGF1R-deficient female mice (Mao et al., 2018; Bokov et al., 2011). To investigate the link between EI24 and aging, we analyzed genotype-tissue expression (GTEx) data on EI24 mRNA levels in muscle tissue across age groups (Consortium, 2013), which revealed lower expression in older groups compared to younger groups (Supplementary Figure S2), prompting further exploration of how elevated EI24 levels might impact aging. Kaplan–Meier survival analysis revealed that male *Ei24* TG mice had relatively longer lifespans than WT mice (Figure 2A), while no significant difference was observed for female mice (Figure 2B). To evaluate cellular aging, SA-β-gal staining was performed on the kidneys, liver, and pancreas of 75-week-old WT and *Ei24* TG mice (Figure 2C). Quantification of the staining intensities revealed a significant reduction in SA-β-gal staining in the *Ei24* TG mice (Figures 2D–F), suggesting that Ei24 may play a role in alleviating cellular aging.

### 3.3 Ei24 overexpression improves glucose tolerance

**FIGURE 3:**
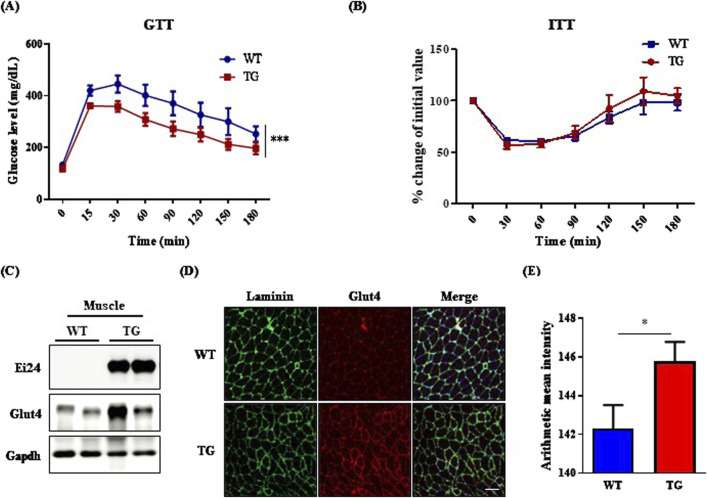
*Ei24* overexpression enhances glucose homeostasis in aged male mice. **(A)** Glucose tolerance test (GTT) results in WT and TG male mice (***p < 0.001, WT n = 12, TG n = 12). **(B)** Insulin tolerance test (ITT) results in WT and TG male mice (WT n = 4, TG n = 5). **(C)** Western blot analysis of endogenous Ei24, Glut4, and Gapdh in the muscle tissues of WT and TG male mice (WT n = 2, TG n = 2). **(D)** Immunofluorescence of Glut4 (red) and Laminin (green) in the muscle tissues of WT and TG male mice. DAPI-stained nuclei (blue; scale bar = 100 µm). **(E)** Quantification of Glut4 fluorescence intensity in muscle tissues (*p < 0.05, WT n = 6, TG n = 6).

IGF1R depletion in muscle increases Glut4 transcription, improving metabolic regulation and glucose uptake (O'Neill et al., 2015). Middle-aged IGF1R-deficient male mice also resist aging and the effects of a high-fat diet on adiposity and glucose metabolism (Perez-Matute et al., 2022). Building on these studies, we examined the impact of *Ei24* overexpression on glucose homeostasis in aged mice. The GTT revealed that *Ei24* TG mice had significantly enhanced glucose tolerance, with lower blood glucose levels than WT mice post administration (Figure 3A). However, the ITT revealed no significant differences in insulin sensitivity between the groups (Figure 3B). The improved glucose tolerance in TG mice appears to be driven by enhanced Glut4 expression in the muscle, as confirmed by Western blotting and immunofluorescence assay (Figures 3C–E). These findings suggest that elevated glucose uptake, facilitated by higher Glut4 levels, underlies the metabolic benefits of aging. Furthermore, Ei24 was strongly correlated with muscle differentiation, as shown by the upregulation of the myoblast differentiation markers, *Myf5*, *Pgc-1α*, and *Ckm*, indicating that Ei24 might enhance muscle function and metabolic health in aging (Supplementary Figure S3) (Zhang et al., 2020; Hu et al., 2023).

### 3.4 Ei24 overexpression confers resistance to STZ-induced diabetes

**FIGURE 4:**
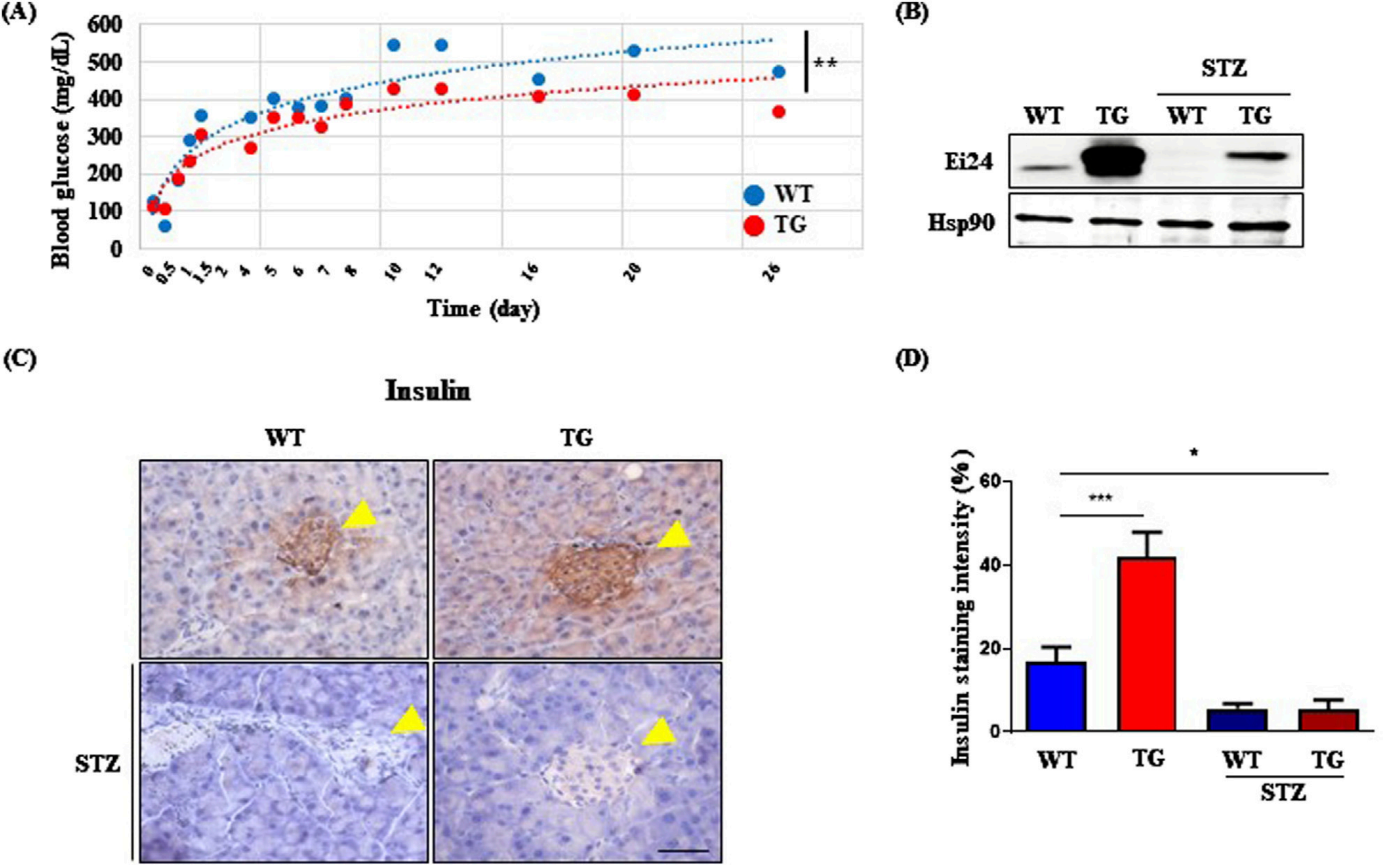
*Ei24* overexpression confers resistance to streptozotocin (STZ)-induced diabetes. **(A)** Blood glucose levels of 8-week-old WT and *Ei24* TG mice after STZ administration (**p < 0.01, WT n = 6, TG n = 6). **(B)** Western blot analysis of Ei24 and Hsp90 in the pancreas of WT and TG mice, both with and without STZ treatment. **(C)** Immunohistochemistry (IHC) for insulin (brown) in the pancreas of WT and TG mice. Yellow arrowheads indicate islets of the pancreas. Hematoxylin-stained nuclei (blue-purple; scale bar = 50 µm). **(D)** Quantification of insulin staining intensity in pancreatic islets (*p < 0.05, ***p < 0.001, WT n = 8, TG n = 24, STZ-treated WT n = 9, STZ-treated TG n = 22).

Building on these findings, we hypothesized that the EI24-IGF1R interaction could alleviate DM by improving metabolic function. To test this hypothesis, we investigated the impact of *Ei24* overexpression in mitigating streptozotocin (STZ)-induced diabetes, which mimics β-cell destruction. After STZ administration, TG mice exhibited significantly lower blood glucose levels compared to WT mice, indicating a protective effect of Ei24 against hyperglycemia (Figure 4A). Given that Ei24 enhances β-cell survival by regulating Atp2a2 in β-cell–specific *Ei24* knockout mice (Yuan et al., 2018), the expression of Ei24 in β-cells is essential. Western blot analysis showed elevated Ei24 protein levels in the TG mice pancreas compared with WT controls, which declined following STZ treatment (Figure 4B). Because this analysis was performed using whole pancreas lysates, the observed protein signal may reflect Ei24 expression in other pancreatic cell types beyond β-cells. In addition, the reduction in Ei24 protein levels post-STZ administration can possibly be attributed to proteasomal and autophagy-mediated degradation induced by reactive oxygen species generated during STZ exposure (Gonzalez et al., 2011; Kaniuk et al., 2007; Xu et al., 2022). Further support for the protective role of Ei24 came from the insulin immunohistochemistry, which revealed a higher intensity of insulin staining in the pancreas of TG mice compared with WT mice under normal conditions (Figure 4C). However, after STZ treatment, the percentage of insulin-positive cells in both groups were similar (Figure 4D), suggesting that Glut4 activation in the muscle may have helped mitigate severe DM symptoms by enhancing glucose uptake and reducing hyperglycemia.

## 4 Discussion

The results of this study elucidate the multifaceted roles of EI24 in regulating various cellular processes, including cancer proliferation, autophagy, and homeostasis (Xu et al., 2022; Devkota et al., 2012; Zang et al., 2018; Devkota et al., 2016). We identified an interaction between EI24 and IGF1R, suggesting a potential functional link (Figure 1A). IGF1R is known to modulate cell growth, proliferation, metabolism, and aging-related biological processes (Barclay et al., 2019; Vitale et al., 2019; LeRoith et al., 2021). Upon ligand binding, IGF1R undergoes conformational changes and autophosphorylation, which are essential for its activity (Jamwal et al., 2018; Li et al., 2019). Our findings revealed that EI24 binds to the TM domain of IGF1R and regulates its phosphorylation (Figures 1C,E; Supplementary Figures S1B, C). Given that reduced Igf1r signaling is linked to improved longevity and metabolic regulation (Azpurua et al., 2013; Gubbi et al., 2018; Mao et al., 2018), Ei24 overexpression may mimic the phenotypes observed in Igf1r knockout mice by inhibiting IGF1R phosphorylation.

In terms of longevity, *Ei24* TG mice displayed a modest increase in overall survival compared with WT mice (Figure 2A), likely due to the autophagic functions of Ei24 (Devkota et al., 2016). Lifespan extension via autophagy induction has been documented in mouse models overexpressing *Atg* or harboring *Beclin1* mutations (Pyo et al., 2013; Fernandez et al., 2018). Interestingly, the beneficial effects of Ei24 overexpression on aging appeared to be male-specific, as female mice showed no such improvement (Figure 2A), suggesting that the Ei24-IGF1R interaction may underlie this sex-specific aging phenotype.

Sirt6 TG male mice, which exhibit Igf1r inhibition, demonstrate improved glucose tolerance and extended lifespan, effects that are absent in female mice (Kanfi et al., 2012; Tang and Fan, 2019; Samant et al., 2021). Conversely, Igf1r suppression in female mice confers a modest lifespan increase, while in middle-aged male mice, it enhances metabolic homeostasis without extending the lifespan (Mao et al., 2018; Perez-Matute et al., 2022). These results suggest a sex-specific response (Bokov et al., 2011; Mao et al., 2018), highlighting the need for further Igf1r-related aging research. *Ei24* TG mice exhibited superior glucose tolerance, likely due to increased Glut4 expression in aged muscle (Figures 3C–E). In addition, Ei24 exhibited a strong correlation with muscle differentiation, as indicated by the upregulation of markers such as *Myf5*, *Pgc-1α*, and *Ckm* (Supplementary Figure S3). These observations support a link between Ei24 and Igf1r signaling, which is essential for muscle growth and differentiation (O'Neill et al., 2015; Barclay et al., 2019), with *Ei24* overexpression in males potentially promoting healthy aging via distinct metabolic pathways.

Blocking Igf1r in β-cells can mitigate aging-induced β-cell senescence and dysfunction, which contribute to a diabetes-related decline in β-cell function (Iwasaki et al., 2023; Perez-Matute et al., 2022). Before STZ treatment, *Ei24* TG mice exhibited a significant induction in insulin levels compared with WT mice (Figure 4D), suggesting that *Ei24* overexpression in young mice enhances insulin protein expression by suppressing IGF1R signaling. However, in older mice, Ei24 did not affect the ITT results (Figure 3B), a finding which aligns with the those observed in Igf1r-deficient young and middle-aged mice (Perez-Matute et al., 2022). Our results implied that in β-cells, Ei24 may interact with IGF1R to regulate blood glucose levels in young mice.

In summary, Ei24 overexpression promotes autophagy, improves glucose homeostasis through increased Glut4 expression, and contributes to healthier aging. These findings provide new insights into the dual role of Ei24 in regulating both autophagy and Igf1r signaling, suggesting potential therapeutic applications for metabolic and age-related diseases. Future studies should aim to elucidate the molecular pathways through which Ei24 exerts its effects and explore its potential in clinical settings for managing metabolic disorders and promoting healthy aging.

## Data Availability

The raw data supporting the conclusions of this article will be made available by the authors, without undue reservation.
