# Supplementary figures and images for: EI24 binds to IGF1R, enhancing glucose homeostasis and fostering healthy aging in male mice
Source: Front Aging. 2025 Jun 10;6:1564730. doi: 10.3389/fragi.2025.1564730 (PMC12185461; doi:10.3389/fragi.2025.1564730)

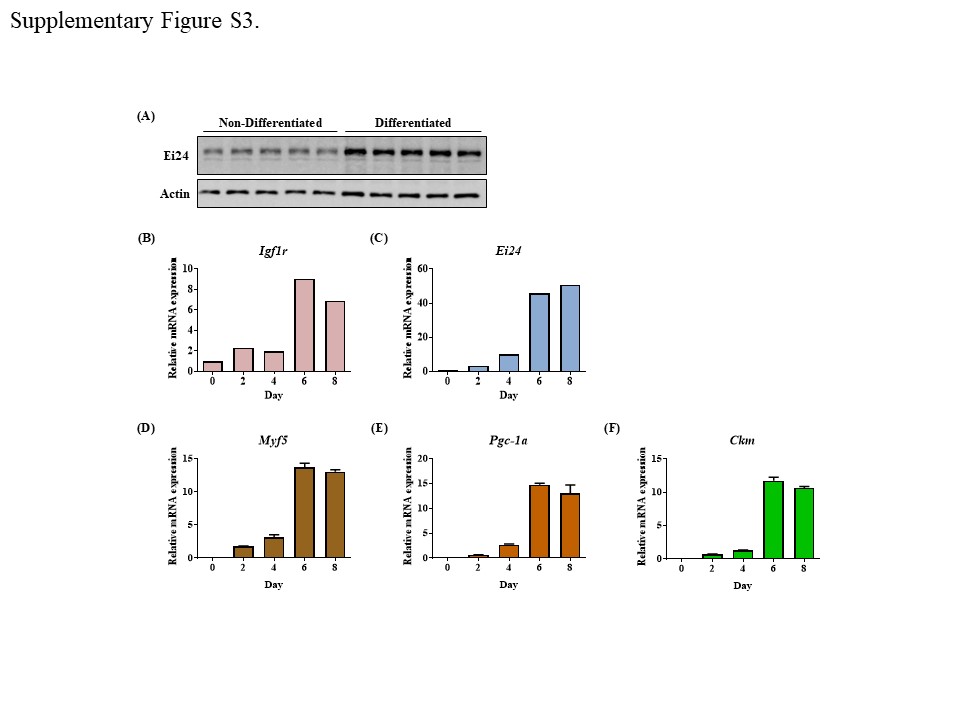

Supplement: Supplementary file 1 [file Image3.jpeg]

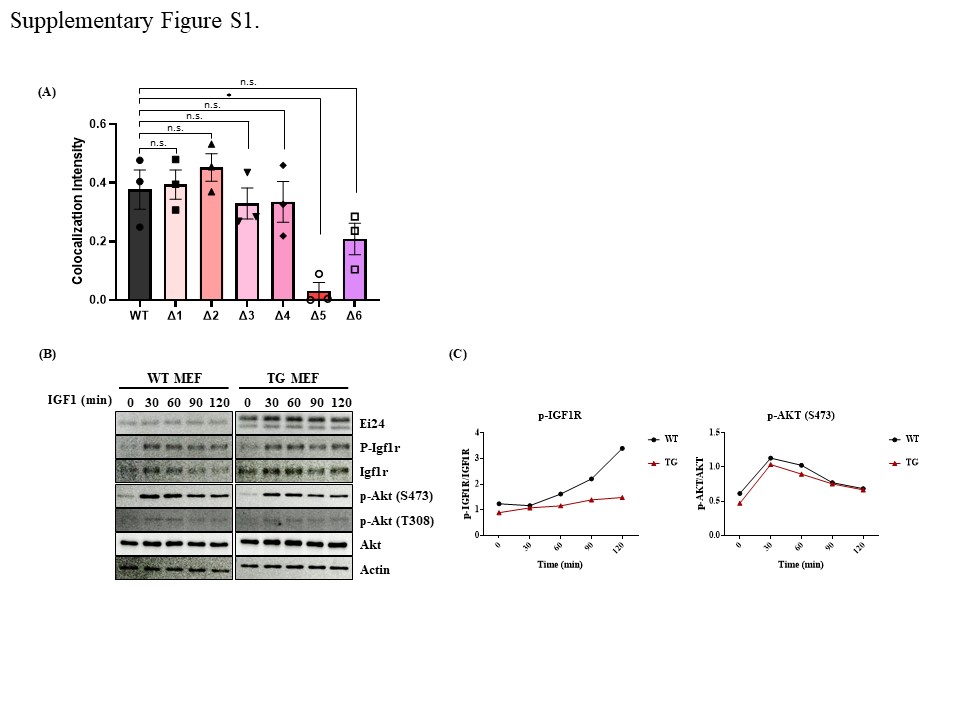

Supplement: Supplementary file 3 [file Image1.jpeg]

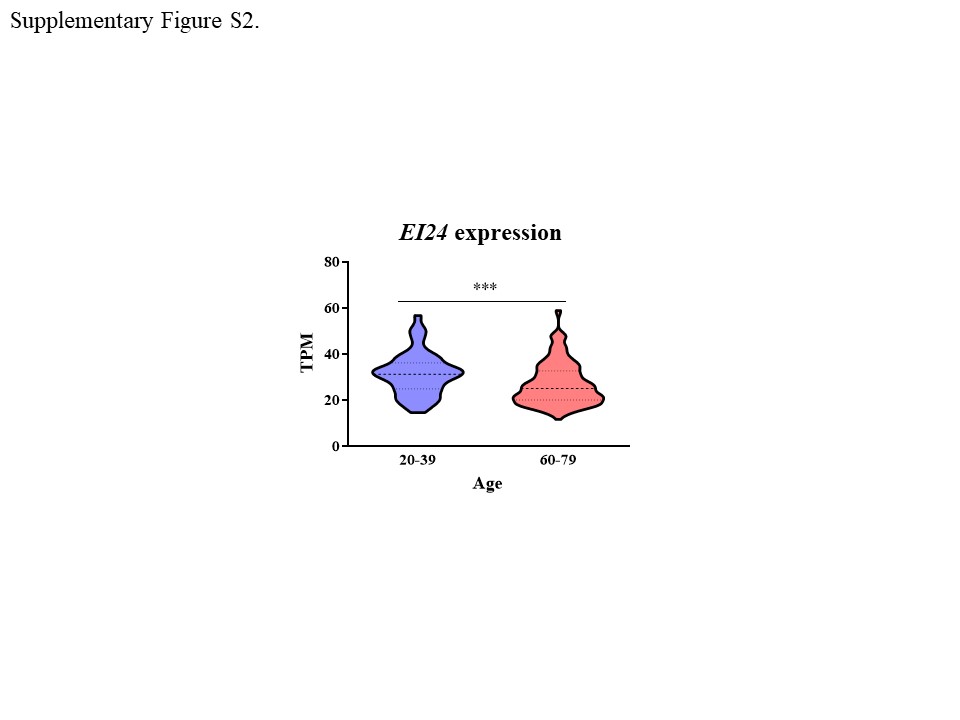

Supplement: Supplementary file 4 [file Image2.jpeg]
