# Supplementary material for: EI24 binds to IGF1R, enhancing glucose homeostasis and fostering healthy aging in male mice
Source: Front Aging. 2025 Jun 10;6:1564730. doi: 10.3389/fragi.2025.1564730 (PMC12185461; doi:10.3389/fragi.2025.1564730)
Supplement: Supplementary file 2 [file Table1.docx]

**Ei24 binds to IGF1R, enhancing glucose homeostasis and fostering healthy aging in male mice.**

**Supplementary data and material**

**Figure S1. Phosphorylation of IGF1R reduce in EI24 TG MEF.**

(A) Quantification of the colocalization of EI24 and IGF1R signals in 293T cells of Fig. 1D (*p < 0.05). (B) Western blot analysis of time-course analysis of IGF1-induced IGF1R phosphorylation in wild-type (WT) and Ei24 TG MEFs. Prior to IGF1 treatment, both MEF lines were serum-starved for 24 hours. IGF1 (100 ng/mL) was then added to both groups. (C) Quantification of phosphorylated IGF1R relative to total IGF1R, and phosphorylated AKT (S473) relative to total AKT.

**Figure S2. Expression of EI24 decreases by aging in GTEx Dataset.**

The expression levels of EI24 in muscle of two age groups: 20-30 years (n=132) and 60-70 years (n=292). The analysis revealed a significant reduction in EI24 expression in the 60-70 years age group compared to the 20-30 years group (***p < 0.001).

**Figure S3. Ei24 has a correlation with the C2C12 myoblast differentiation.**

(A) Western blot analysis of Ei24 expressions in C2C12 myoblasts after 10 days of muscle differentiation. The samples were prepared from five different wells to ensure accuracy. Actin is shown as a loading control. (B-F) Relative mRNA expression of Igf1r, Ei24, Myf5, Pgc-1α, and Ckm during C2C12 differentiation, measured by quantitative RT-PCR (Igf1r and Ei24: n=1 each, Myf5, Pgc-1α, and Ckm: n=2 each).

**Table S1. List of the antibodies used in the study.**

| **Antibody** | **Manufacture** | **Catalog No.** | **Dilution^a^** |
| --- | --- | --- | --- |
| EI24 | Atlas antibodies  (Stockholm, SE) | HPA047165 | WB, 1:1000  IF, 1:100 |
| GLUT4 | Abcam  (Cambridge, UK) | Ab34088 | WB, 1:1000  IF, 1:100 |
| GLUT4 | Santa Cruz Biotechnology  (Dallas, TX) | sc-53566 | WB, 1:1000 |
| IGF1R | Cell Signaling Technology  (Beverly, MA) | #9750 | WB, 1:1000 |
| IGF1R | Santa Cruz Biotechnology  (Dallas, TX) | sc-712 | WB, 1:1000 |
| IGF1R | Santa Cruz Biotechnology  (Dallas, TX) | sc-713 | WB, 1:1000 |
| Hsp90α/β | Santa Cruz Biotechnology  (Dallas, TX) | sc-13119 | WB, 1:1000 |
| ꞵ-Actin | Santa Cruz Biotechnology  (Dallas, TX) | sc-517582 | WB, 1:1000 |
| HA | Santa Cruz Biotechnology  (Dallas, TX) | sc-7392 | WB, 1:1000  co-IP |
| Flag | Santa Cruz Biotechnology  (Dallas, TX) | sc‑166355 | WB, 1:1000  IF, 1:100  co-IP |
| c-Myc | Santa Cruz Biotechnology  (Dallas, TX) | sc-40 | WB, 1:1000 |
| c-Myc | Cell Signaling Technology  (Beverly, MA) | #5605 | IF, 1:100 |
| Laminin | Santa Cruz Biotechnology  (Dallas, TX) | sc-55605 | IHC, 1:100 |
| Insulin | Cell Signaling Technology  (Beverly, MA) | #3014 | IHC, 1:100 |
| Goat anti-mouse (H+L)-HRP | GenDEPOT  (Katy, TX ) | SA001 | WB, 1:10000  IHC, 1:100 |
| Goat anti-rabbit (H+L)-HRP | GenDEPOT  (Katy, TX ) | SA002 | WB, 1:10000  IHC, 1:100 |
| Anti-rabbit IgG – Alexa 594 | Invitrogen  (Carlsbad, CA) | A-21207 | IHC, 1:100  IF, 1:100 |
| Anti-mouse IgG – Alexa 488 | Invitrogen  (Carlsbad, CA) | A-11001 | IHC, 1:100  IF, 1:100 |
| Anti-rabbit IgG – Alexa 488 | Invitrogen  (Carlsbad, CA) | A-21206 | IHC, 1:100  IF, 1:100 |
| Anti-mouse IgG –Alexa 594 | Invitrogen  (Carlsbad, CA) | A-32744 | IHC, 1:100  IF, 1:100 |

^a^WB, western blot; IF, immunofluorescence; IHC, immunohistochemistry; IP, immunoprecipitation;

**Table S2. List of the QPCR primers used in the study.**

| **Primers** | **Sequence（5’-3’）** |
| --- | --- |
| *Igf1r*-F | GCTTCGTTATCCACGACGATG |
| *Igf1r* -R | GAATGGCGGATCTTCACGTAG |
| *Ei24*-F | GACCTTGGCAGGGGAATCAA |
| *Ei24*-R | GCTTCCGCTCTACACTCTGG |
| *Gapdh*-F | TTCACCACCATGGAGAAGGC |
| *Gapdh*-R | CCCTTTTGGCTCCACCCT |
| *Myf5*-F | CAGCCCCACCTCCAACTG |
| *Myf5*-R | GCAGCACATGCATTTGATACATC |
| *Pgc-1α-*F | TGCAGCGGTCTTAGCACTCA |
| *Pgc-1α*-R | CATGAATTCTCGGTCTTAACAATGG |
| *Creatine kinase muscle* (*Ckm*)-F | GCACTGGCCGCAGCAT |
| *Creatine kinase muscle* (*Ckm*)-R | GAGGGTAGTACTTGCCCTTGAACTC |
